# Association between haemoglobin A1c and all-cause and cause-specific mortality in middle-aged and older Koreans: a prospective cohort study

**DOI:** 10.1186/s12986-022-00682-4

**Published:** 2022-07-14

**Authors:** Bo Mi Song, Jung Hyun Lee, Hae Dong Woo, Mi Jin Cho, Sung Soo Kim

**Affiliations:** 1 Division of Population Health Research, Department of Precision Medicine, Korea National Institute of Health, Korea Disease Control and Prevention Agency, Cheongju, Chungcheongbuk-do South Korea; 2 Department of Chronic Disease Convergence Research, Korea National Institute of Health, Korea Disease Control and Prevention Agency, Cheongju, Chungcheongbuk-do South Korea

**Keywords:** All-cause mortality, Cardiovascular mortality, Cancer mortality, Cause-specific mortality, Glycated haemoglobin, Haemoglobin A1c, Korean, Liver diseases, Red blood cell, Time-dependent Cox proportional hazards model

## Abstract

**Background:**

This study aimed to examine associations between haemoglobin A1c (HbA1c) levels over time and all-cause and cause-specific mortality in middle-aged and older Koreans.

**Methods:**

Using 16 years of follow-up data from the Korean Genome and Epidemiology Study, we analysed 9294 individuals aged 40–69 years with no history of cardiovascular disease (CVD) or cancer. Participants were divided into a known diabetes group and five groups categorized by HbA1c levels (< 5.0%, 5.0–5.4%, 5.5–5.9%, 6.0–6.4%, and ≥ 6.5%). Hazard ratios (HRs) for all-cause and cause-specific mortality associated with HbA1c levels were calculated using a conventional and a time-dependent Cox proportional hazards model. Restricted cubic spline models were fitted to investigate the relationship between continuous HbA1c levels and mortality among people without known diabetes. Subgroup analyses were performed for age, sex, smoking, hypertension, liver diseases, and red blood cell counts.

**Results:**

During a median follow-up period of 15.7 years, there were 944 deaths, including 185 deaths from CVD, 359 from cancer, and 125 from all external causes. Compared with participants with HbA1c levels of 5.5–5.9%, multivariate-adjusted HRs and 95% confidence intervals for all-cause death of participants with levels < 5.0%, 5.0–5.4%, 6.0–6.4%, and ≥ 6.5% and participants with known diabetes were 1.84 (1.35–2.51), 1.13 (0.95–1.34), 1.30 (1.04–1.62), 1.37 (0.97–1.93), and 2.03 (1.70–2.44), respectively. The risk of cancer mortality was significantly increased in HbA1c < 5.0% (HR, 2.21; 95% CI 1.42–3.44) and known diabetes (HR, 1.60; 95% CI 1.18–2.15). When we performed diverse subgroup analyses, low HbA1c levels at baseline were strongly associated with mortality in participants with liver diseases.

**Conclusions:**

We found U-shaped associations between HbA1c levels at baseline and over time and all-cause mortality in middle-aged and older Koreans. Additionally, the risk of cancer mortality increased both in low and high HbA1c groups, but CVD mortality increased only in high HbA1c group. In particular, people with liver diseases and low HbA1c levels had a high risk of all-cause mortality. Therefore, more careful management of these groups is suggested to identify any deteriorating health conditions.

**Graphical abstract:**

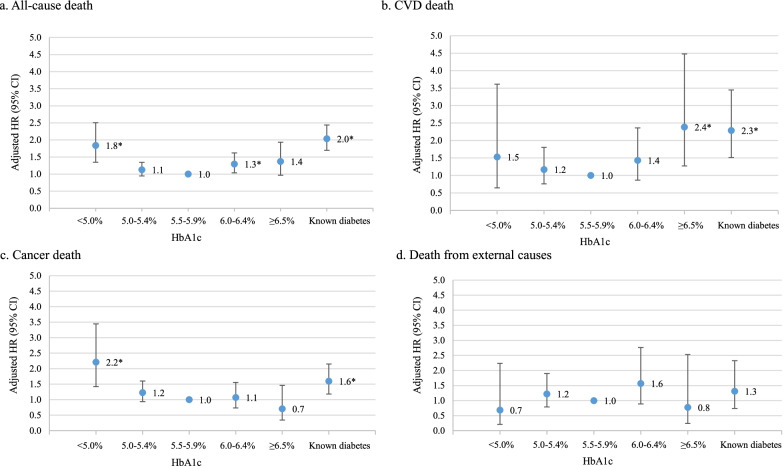

**Supplementary Information:**

The online version contains supplementary material available at 10.1186/s12986-022-00682-4.

## Background

The prevalence of diabetes, a major cause of premature death and disability, is increasing worldwide, especially in Asia [1–3]. Glycated haemoglobin (HbA1c) represents the average glycemia for the preceding 2–3 months and is an established biomarker for monitoring glycaemic control in patients with diabetes [4]. Compared with fasting blood glucose, HbA1c has the advantages that it provides higher repeatability and can be assessed in the non-fasting state [4, 5]. In 2010, the American Diabetes Association and the World Health Organization added an HbA1c threshold of ≥ 6.5% as a standard criterion for the diagnosis of diabetes [6, 7].

In previous studies, the relationship between HbA1c levels and mortality was conflicting. Some cohort studies have observed a positive linear relationship between HbA1c levels and mortality [8–11], whereas others have reported U- or J-shaped associations, with increased mortality at both low and high HbA1c levels [12–16]. This inconsistency might be due to differences in the characteristics of the study populations, causes of death, control for important confounders, and analytic methods. Most of the studies on the association between HbA1c levels and mortality were conducted in western countries, with only a few exceptions targeting the Asian population [9, 17]. Since racial differences have also been reported that HbA1c levels in Asians are higher than in Whites among individuals without diabetes [18], patients with impaired glucose tolerance [19], and patients with type 2 diabetes [20], it is necessary to study the relationship between HbA1c levels and mortality in Asians. Moreover, most of the previous studies used a single HbA1c measurement; therefore, could not reflect the change in glycemic status during follow up. Therefore, we examined whether HbA1c was associated with all-cause and cause-specific mortality using repeated measures from 16 years of cohort follow up data in middle-aged and older Koreans.

## Methods

### Study population

**Fig. 1:**
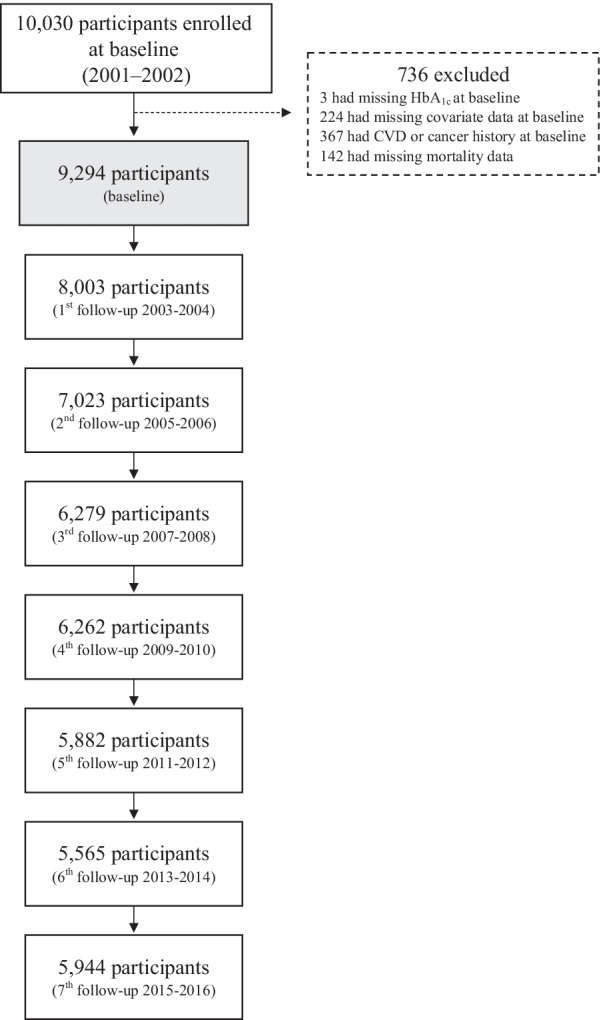
Flow chart of the cohort study

We used data from the Ansan–Ansung Cohort of the Korean Genome and Epidemiology Study (KoGES), which is an ongoing prospective community-based cohort study investigating the environmental and genetic factors affecting prevalent chronic diseases. The study design is described in detail elsewhere [21]. In brief, the Ansan–Ansung cohort study recruited 10,030 individuals aged 40–69 years in a rural (Ansung) and an urban (Ansan) area from 2001 to 2002. Participants were followed up biennially, and we used the follow-up data until 2016 for this study. The final analytical sample of the present study consisted of 9294 participants (4458 men and 4836 women) after excluding those without HbA1c data (n = 3), those with missing covariates (n = 224), those having a history of CVD or cancer (n = 367) at baseline, and those without linked mortality data (n = 142) (Fig. 1). All participants provided written informed consent, and the study protocol was approved by the Institutional Review Board of National Institute of Health, Korea.

### Measurements

During each examination, information on socio-demographic and lifestyle characteristics, personal medical histories and medication usage were collected by trained interviewers using structured questionnaires.

Anthropometric measurements were obtained by trained research staff using standardized protocols and all measuring instruments were calibrated everyday before examination. Standing height was measured using a stadiometer (SECA 225; SECA, Germany) and body weight was measured using a digital scale (GL-60000-20; CAS Korea, Korea). Waist circumference was measured at the midpoint between the lower rib margin and the top of the iliac crest in the standing position. Blood pressure was measured by nurses in both arms in a sitting position with the arms supported at heart level after 5 min of rest using mercury sphygmomanometers (Baumanometer-Standby; W.A. Baum Co. Inc., USA). We used means of measurements from both arms for systolic blood pressure (SBP) and diastolic blood pressure (DBP).

Blood samples of all participants were collected from the antecubital vein after at least 8 h of fasting. HbA1c levels were measured using high performance liquid chromatography (Bio-Rad Variant II; Bio-Rad Laboratories, Inc., Japan) according to the National Glycohemoglobin Standardization Program. The total cholesterol, high-density lipoprotein (HDL) cholesterol, triglycerides, fasting blood glucose, total bilirubin, aspartate aminotransferase (AST), and alanine aminotransferase (ALT) levels were measured using enzymatic methods (HITACHI Automatic Analyzer 7600; Hitachi, Japan and ADVIA 1650 Auto Analyzer; Siemens, USA). The red blood cell (RBC), hemoglobin, and hematocrit levels were measured using optical methods by laser beam (ADVIA 120 Hematology System; BAYER, USA).

Known diabetes was defined as having a history of physician-diagnosed diabetes or under treatment with oral antidiabetic agents or insulin at each wave of examination. Hypertension was defined as SBP ≥ 140 mmHg or DBP ≥ 90 mmHg, having a history of physician-diagnosed hypertension, or under treatment for hypertension. Dyslipidemia was defined as total cholesterol ≥ 230 mg/dL, HDL cholesterol < 40 mg/dL, or triglycerides ≥ 200 mg/dL, having a history of physician-diagnosed dyslipidemia, or under treatment for dyslipidemia. Liver disease was defined as having a history of physician-diagnosed hepatitis or liver cirrhosis. Anaemia was defined as haemoglobin < 13 g/dL for men and haemoglobin < 12 g/dL for women.

### Mortality outcomes

The cohort data were linked to death records (until 31 December 2017) provided by Statistics Korea using resident registration numbers, a unique identifier assigned to all citizens in the Republic of Korea. All death certificates in Koreans are registered to the government, and cause-of-death are ascertained by medical certificates supplemented by linkage to 21 administrative datasets (e.g. National Health Insurance data, National Cancer Registry, and Police Report) to enhance acccuracy of identifying the underlying causes by Statistic Korea (Statistics Korea, Annual report on the causes of death statistics, 2021). The present study investigated the all-cause mortality and cause-specific mortality from CVD (I00 to I99) and cancer (C00 to C97) as classified by the Korean Classification of Diseases, 6th Revision (the Korean version of the International Classification of Diseases, 10th Revision). We also examined mortality from all external causes (S00 toT98) as a negative control.

### Statistical analyses

Participants were divided into a known diabetes group and five groups categorized by HbA1c levels (< 5.0%, 5.0–5.4%, 5.5–5.9%, 6.0–6.4%, and ≥ 6.5%) as previously described [9, 12, 22]. The baseline characteristics of participants across HbA1c groups are presented as means and standard deviations for normally distributed continuous variables and as medians and interquartile ranges for skewed continuous variables. Categorical variables are presented as numbers with percentages. Linear trends across HbA1c groups were tested using linear regression for continuous variables and the Cochran–Armitage test or the Mantel–Haenszel test for categorical variables. The Cox proportional hazards model was used to calculate hazard ratios (HRs) for all-cause and cause-specific mortality according to HbA1c category. The proportional hazards assumption was assessed by using log–log survival plots, and no violations were detected. The 5.5–5.9% HbA1c category was set as the reference group. Covariates consisted of factors affecting HbA1c levels and factors reported in the literature to be related to both HbA1c levels and mortality: age (years), sex, residential area (Ansung or Ansan), BMI (kg/m^2^), smoking (current, past, or never), alcohol use (current, past, or never), regular exercise (yes or no), education (above or below university), hypertension (yes or no), and dyslipidaemia (yes or no).

Models were constructed in the following two ways: (1) a standard Cox proportional hazards model using the baseline HbA1c levels and covariates and (2) a time-dependent Cox propotional hazards model considering HbA1c levels, age, BMI, smoking, alcohol use, regular exercise, hypertension, and dyslipidemia that change over time as time-dependent variables using information from follow-up examination. Missing data were replaced with the values measured in the previous examination. When estimating the risk of cause-specific mortality from CVD, cancer, and external causes, mortality from the other causes was considered competing risk using the Fine–Grey model. Person-years for each participant were calculated as the duration from the baseline examination date to the date of death or 31 December 2017 depending on which came first. Mortality rates per 1000 person-years were calculated for each HbA1c category.

To assess whether the association between HbA1c levels and the risk of mortality differed according to the characteristics of study participants, subgroup analyses were performed for age, sex, smoking status, hypertension, liver diseases, and RBC count. We also tested the interaction effect between HbA1c levels and the characteristics of study participants on mortality using interaction terms.

For sensitivity analysis, we additionally adjusted for RBC count, haemoglobin level, anaemia, and liver diseases which are known to affect HbA1c in our analytical models. We also adjusted for waist circumference instead of BMI. Furthermore, to rule out the effects of possible subclinical disease or underlying poor health condition, we excluded 77 people who died within the first 2 years of follow-up.

After excluding participants with known diabetes, restricted cubic spline regression analyses were performed to model the shape of the association between continuous HbA1c levels at baseline and mortality. Knots were set at the 5th, 25th, 75th, and 95th percentiles and reference was set at the median HbA1c level of 5.6%. The plot was truncated at the 1st and 99th percentiles.

Additionally, we compared the receiver-operating characteristic (ROC) curves and the area under the curve (AUC) for mortality of a conventional model and the model plus baseline HbA1c. The conventional model included age, sex, residential area, BMI, smoking, alcohol use, regular exercise, education, hypertension, and dyslipidemia.

SAS version 9.4 (SAS Institute, Cary, NC, USA) was used for all statistical analyses, and statistical significance was defined as two-tailed *p* values < 0.05.

## Results

Table 1 presents the baseline characteristics of the study population stratified by HbA1c levels. Participants with higher HbA1c levels were more likely to be older and had higher BMI, waist circumferences, RBC counts, haemoglobin levels, hypertension and dyslipidaemia prevalences, and proportion of current smokers; lower prevalence of anaemia, proportion of current alcohol drinkers, and education level. On the other hand, participants with lower HbA1c levels tended to have lower RBC counts and higher prevalence of anaemia and liver diseases.

**Table 1:** Baseline characteristics of study participants according to HbA1c levels at baseline

Variables	Total (*N* = 9294)	HbA1c in participants without known diabetes					*p* trend	Known diabetes (*n* = 604)
		< 5.0%	5.0–5.4%	5.5–5.9%	6.0–6.4%	≥ 6.5%		
		(*n* = 303)	(*n* = 2962)	(*n* = 3930)	(*n* = 1038)	(*n* = 457)		
Age, years	52.0 ± 8.9	48.5 ± 7.6	49.5 ± 8.2	52.4 ± 8.9	54.6 ± 8.5	55.2 ± 8.8	< 0.001	56.5 ± 8.6
Men	4458 (48.0)	132 (43.6)	1387 (46.8)	1904 (48.5)	498 (48.0)	211 (46.2)	0.362	326 (54.0)
Body mass index, kg/m^2^	24.6 ± 3.1	23.8 ± 2.8	23.9 ± 2.9	24.6 ± 3.1	25.5 ± 3.1	26.3 ± 3.3	< 0.001	25.3 ± 3.1
Waist circumference, cm	82.6 ± 8.8	80.0 ± 8.5	80.0 ± 8.5	82.6 ± 8.6	85.7 ± 8.3	88.5 ± 7.9	< 0.001	86.5 ± 7.9
Fasting Glucose, mg/dL	82 [77, 90]	79 [74, 84]	80 [76, 85]	82 [77, 89]	87 [81, 95]	108 [94, 130]	< 0.001	119 [94, 158]
HbA1c, %	5.8 ± 0.9	4.8 ± 0.3	5.3 ± 0.1	5.7 ± 0.1	6.1 ± 0.1	7.4 ± 1.2	< 0.001	7.9 ± 1.9
RBC count, Mil/µL	4.42 ± 0.47	4.32 ± 0.52	4.38 ± 0.47	4.42 ± 0.46	4.47 ± 0.46	4.53 ± 0.46	< 0.001	4.49 ± 0.50
Haemoglobin, g/dL	13.6 ± 1.6	13.5 ± 1.7	13.6 ± 1.6	13.6 ± 1.6	13.6 ± 1.6	13.8 ± 1.5	0.063	13.8 ± 1.6
Haematocrit, %	41.0 ± 4.6	40.4 ± 4.9	40.9 ± 4.5	41.0 ± 4.6	41.2 ± 4.7	41.7 ± 4.3	< 0.001	41.7 ± 4.7
Total bilirubin, mg/dL	0.53 [0.40, 0.73]	0.64 [0.48, 0.92]	0.57 [0.43, 0.78]	0.52 [0.38, 0.69]	0.49 [0.37, 0.66]	0.50 [0.39, 0.71]	< 0.001	0.50 [0.38, 0.69]
ALT, IU/L	22 [17, 31]	21 [16, 29]	21 [16, 28]	22 [18, 31]	25 [19, 34]	30 [22, 44]	< 0.001	27 [20, 36]
AST, IU/L	26 [23, 32]	25 [21, 32]	26 [22, 31]	27 [23, 32]	27 [24, 32]	29 [24, 38]	< 0.001	26 [22, 33]
Systolic blood pressure, mmHg	121.3 ± 18.4	117.8 ± 17.7	118.0 ± 17.6	121.3 ± 18.2	124.5 ± 18.4	129.5 ± 19.2	< 0.001	127.8 ± 18.9
Diastolic blood pressure, mmHg	80.2 ± 11.4	78.2 ± 12.2	78.6 ± 11.4	80.5 ± 11.4	81.8 ± 11.3	84.5 ± 10.7	< 0.001	81.7 ± 10.7
Hypertension	2770 (29.8)	76 (25.1)	693 (23.4)	1234 (31.4)	403 (38.8)	219 (47.9)	< 0.001	314 (52.0)
Dyslipidaemia	4592 (49.4)	103 (34.0)	1204 (40.7)	1977 (50.3)	622 (59.9)	326 (71.3)	< 0.001	403 (66.7)
Liver diseases	411 (4.4)	26 (8.6)	138 (4.7)	147 (3.7)	41 (4.0)	15 (3.3)	0.002	44 (7.3)
Anaemia	1321 (14.2)	47 (15.5)	417 (14.1)	592 (15.1)	139 (13.4)	54 (11.8)	0.271	72 (11.9)
Smoking status							< 0.001	
Never smoker	5450 (58.6)	195 (64.4)	1819 (61.4)	2266 (57.7)	581 (56.0)	265 (58.0)		324 (53.6)
Past smoker	1428 (15.4)	53 (17.5)	457 (15.4)	587 (14.9)	147 (14.2)	58 (12.7)		126 (20.9)
Current smoker	2416 (26.0)	55 (18.2)	686 (23.2)	1077 (27.4)	310 (29.9)	134 (29.3)		154 (25.5)
Alcohol use							< 0.001	
Never alcohol drinker	4262 (45.9)	125 (41.3)	1316 (44.4)	1819 (46.3)	480 (46.2)	230 (50.3)		292 (48.3)
Past alcohol drinker	589 (6.3)	18 (5.9)	144 (4.9)	257 (6.5)	77 (7.4)	34 (7.4)		59 (9.8)
Current alcohol drinker	4443 (47.8)	160 (52.8)	1502 (50.7)	1854 (47.2)	481 (46.3)	193 (42.2)		253 (41.9)
Regular exercise	2613 (28.1)	81 (26.7)	875 (29.5)	1065 (27.1)	251 (24.2)	130 (28.5)	0.031	211 (34.9)
Higher education	1254 (13.5)	44 (14.5)	440 (14.9)	526 (13.4)	113 (10.9)	54 (11.8)	< 0.001	77 (12.8)
Residential area								
Ansung	4532 (48.8)	127 (41.9)	1289 (43.5)	1968 (50.1)	557 (53.7)	253 (55.4)	< 0.001	338 (56.0)
Ansan	4762 (51.2)	176 (58.1)	1673 (56.5)	1962 (49.9)	481 (46.3)	204 (44.6)		266 (44.0)

*p* for trend was derived from a general linear model using contrast coefficients, the Cochran–Armitage trend test or the Mantel–Haenszel test

Known diabetes: a history of physician-diagnosed diabetes or under treatment with oral antidiabetic agents or insulin

Hypertension: SBP ≥ 140 mmHg, DBP ≥ 90 mmHg, history of physician-diagnosed hypertension, or under treatment for hypertension

Dyslipidaemia: total cholesterol ≥ 230 mg/dL, HDL cholesterol < 40 mg/dL, triglycerides ≥ 200 mg/dL, history of physician-diagnosed dyslipidaemia, or under treatment for dyslipidaemia

Liver diseases: history of physician-diagnosed hepatitis or liver cirrhosis

Anaemia: haemoglobin < 13 g/dL for men and haemoglobin < 12 g/dL for women

Regular exercise: exercise at least once a week

Higher education: university graduation or above

During 139,960 person-years of follow-up (median 15.7 years), there were 944 deaths, including 185 deaths from CVD, 359 from cancer, and 125 from external causes. Compared with participants with baseline HbA1c levels of 5.5–5.9% as a reference, multivariate-adjusted HRs and 95% confidence intervals for all-cause mortality of participants with HbA1c levels < 5.0%, 5.0–5.4%, 6.0–6.4%, and ≥ 6.5% and participants with known diabetes were 1.74 (1.18–2.57), 1.20 (1.01–1.42), 1.50 (1.23–1.83), 1.62 (1.24–2.11), and 2.30 (1.88–2.81), respectively. When we considered changes in HbA1c levels and other covariates during follow-up, the U-shaped associations between HbA1c and all-cause mortality persisted. Specifically, significantly elevated risks of mortality were found in HbA1c < 5.0% (HR, 1.84; 95% CI 1.35–2.51), 6.0–6.4% (HR, 1.30; 95% CI 1.04–1.62), and known diabetes (HR, 2.03; 95% CI 1.70–2.44). We also found a U-shaped association for baseline HbA1c with cancer mortality. In a time-dependent Cox model, the association between HbA1c and cancer mortality was not clear except significantly elevated risk in HbA1c < 5.0% (HR, 2.21; 95% CI 1.42–3.44) and known diabetes (HR, 1.60; 95% CI 1.18–2.15). HbA1c levels at baseline examination were not significantly associated with CVD mortality, whereas HbA1c > 6.5% over time showed an elevated risk with HR 2.39 (95% CI 1.27–4.48). Known diabetes assessed at baseline and over time were associated with 130% increased risk of CVD mortality (Table 2).

**Table 2:** Mortality rate and risk of death according to HbA1c levels at baseline or over time

	HbA1c in participants without known diabetes					Known diabetes
	< 5.0%	5.0–5.4%	5.5–5.9%	6.0–6.4%	≥ 6.5%	
*HbA1c at baseline, n*	303	2962	3930	1038	457	604
Person-years of follow-up	4542	44,966	59,625	15,458	6771	8598
All-cause death, *n*	28	228	337	139	69	143
Mortality (per 1000 person-years)	6.2	5.1	5.7	9.0	10.2	16.6
Adjusted HR (95% CI)*	1.74 (1.18–2.57)	1.20 (1.01–1.42)	Ref	1.50 (1.23–1.83)	1.62 (1.24–2.11)	2.30 (1.88–2.81)
CVD death, *n*	3	33	68	30	15	36
Mortality (per 1000 person-years)	0.7	0.7	1.1	1.9	2.2	4.2
Adjusted HR (95% CI)*	1.07 (0.33–3.46)	0.99 (0.65–1.51)	Ref	1.38 (0.89–2.14)	1.41 (0.80–2.48)	2.32 (1.51–3.56)
Cancer death, *n*	12	107	126	52	27	35
Mortality (per 1000 person-years)	2.6	2.4	2.1	3.4	4.0	4.1
Adjusted HR (95% CI)*	1.81 (0.99–3.30)	1.44 (1.11–1.87)	Ref	1.43 (1.03–1.99)	1.65 (1.08–2.52)	1.41 (0.97–2.06)
Death from external causes, *n*	3	38	50	19	6	9
Mortality (per 1000 person-years)	0.7	0.8	0.8	1.2	0.9	1.0
Adjusted HR (95% CI)*	0.94 (0.29–3.08)	1.13 (0.74–1.74)	Ref	1.36 (0.79–2.34)	1.03 (0.43–2.48)	1.01 (0.49–2.06)
HbA1c over time, adjusted HR (95% CI)*			0			
All-cause death,	1.84 (1.35–2.51)	1.13 (0.95–1.34)	Ref	1.30 (1.04–1.62)	1.37 (0.97–1.93)	2.03 (1.70–2.44)
CVD death	1.53 (0.65–3.61)	1.17 (0.76–1.80)	Ref	1.43 (0.87–2.36)	2.39 (1.27–4.48)	2.29 (1.52–3.45)
Cancer death	2.21 (1.42–3.44)	1.23 (0.94–1.60)	Ref	1.07 (0.74–1.56)	0.71 (0.34–1.46)	1.60 (1.18–2.15)
Death from external causes	0.69 (0.21–2.24)	1.22 (0.79–1.90)	Ref	1.57 (0.89–2.76)	0.77 (0.24–2.53)	1.31 (0.74–2.32)

Known diabetes was defined as a history of physician-diagnosed diabetes or under treatment with oral antidiabetic agents or insulin

A standard Cox proportional hazards model was tested using HbA_1c_ at baseline and a time-dependent Cox proportional hazards model was tested using HbA_1c_ over time

^*^Adjusted for age (years), sex, residential area (Ansung or Ansan), body mass index (kg/m^2^), smoking (current, past, or never), alcohol use (current, past, or never), regular exercise (yes or no), education (above or below university), hypertension (yes or no), and dyslipidaemia (yes or no)

The findings were not changed materially after we additionally adjusted for RBC count, haemoglobin level, anaemia, and liver diseases (Additional file 1); excluded 77 people who died within the first 2 years of follow-up (Additional file 2); and adjusted for waist circumference instead of BMI (data not shown).

In subgroup analysis, we observed broadly consistent results for risks of all-cause mortality by baseline HbA1c levels, whereas the risk of all-cause mortality was significantly associated with low HbA1c levels of < 5.0% only among people with liver diseases or below median RBC counts (4.72 Mil/µL for men and 4.15 Mil/µL for women). Interaction was significant only between HbA1c and liver diseases (Table 3).

**Table 3:** Subgroup analyses for risk of all-cause mortality (adjusted HR [95% CI]) according to baseline HbA1c

	No. of	Deaths, *n* (%)	HbA1c in participants without known diabetes					Known diabetes (*n* = 604)	*p*-interaction
			< 5.0%	5.0–5.4%	5.5–5.9%	6.0–6.4%	≥ 6.5%		
			(*n* = 303)	(*n* = 2962)	(*n* = 3930)	(*n* = 1038)	(*n* = 457)		
All-cause death	9294	944 (10.2)	28	228	337	139	69	143	
40–49 years	4497	154 (3.4)	1.37 (0.62–3.04)	1.21 (0.84–1.74)	Ref	1.99 (1.20–3.31)	0.69 (0.21–2.21)	1.30 (0.59–2.86)	0.146
50–69 years	4797	790 (16.5)	1.92 (1.23–3.00)	1.19 (0.98–1.44)	Ref	1.46 (1.17–1.81)	1.73 (1.32–2.27)	2.43 (1.96–3.00)	
Men	4458	603 (13.5)	1.72 (1.07–2.77)	1.20 (0.98–1.48)	Ref	1.64 (1.28–2.11)	1.72 (1.22–2.43)	2.10 (1.62–2.73)	0.424
Women	4836	341 (7.1)	1.76 (0.89–3.49)	1.18 (0.87–1.58)	Ref	1.28 (0.92–1.78)	1.49 (0.99–2.25)	2.71 (1.97–3.73)	
Never smoker	5450	414 (7.6)	1.89 (1.09–3.29)	1.19 (0.91–1.55)	Ref	1.31 (0.95–1.80)	1.87 (1.29–2.70)	2.88 (2.16–3.85)	0.042
Ever smoker	3844	530 (13.8)	1.52 (0.88–2.62)	1.19 (0.96–1.49)	Ref	1.67 (1.29–2.17)	1.45 (0.99–2.12)	1.84 (1.38–2.44)	
Hypertension	2939	429 (14.6)	1.63 (0.88–3.03)	1.25 (0.96–1.65)	Ref	1.49 (1.11–2.00)	1.53 (1.07–2.21)	2.25 (1.70–2.96)	0.967
No hypertension	6355	515 (8.1)	1.79 (1.09–2.96)	1.19 (0.95–1.48)	Ref	1.52 (1.16–2.00)	1.78 (1.22–2.62)	2.39 (1.77–3.21)	
Liver disease	411	65 (15.8)	4.12 (1.51–11.23)	1.73 (0.88–3.40)	Ref	2.51 (1.09–5.76)	2.41 (0.52–11.22)	1.41 (0.63–3.15)	0.036
No liver disease	8883	879 (9.9)	1.43 (0.92–2.24)	1.17 (0.98–1.39)	Ref	1.45 (1.18–1.78)	1.62 (1.24–2.12)	2.34 (1.90–2.88)	
Low RBC	4625	580 (12.5)	2.23 (1.48–3.37)	1.19 (0.97–1.47)	Ref	1.43 (1.10–1.86)	1.40 (0.97–2.04)	2.33 (1.78–3.04)	0.172
High RBC	4669	364 (7.8)	0.42 (0.10–1.72)	1.21 (0.91–1.62)	Ref	1.56 (1.15–2.12)	1.87 (1.28–2.74)	2.21 (1.62–3.01)	

When not stratified by one of the following, models were adjusted for age (years), sex, residential area (Ansung or Ansan), body mass index (kg/m^2^), smoking (current, past, or never), alcohol use (current, past, or never), regular exercise (yes or no), education (above or below university), hypertension (yes or no), and dyslipidaemia (yes or no)

Known diabetes was defined as a history of physician-diagnosed diabetes or under treatment with oral antidiabetic agents or insulin

Low or high RBC was classified based on the median (4.72 Mil/µL for men and 4.15 Mil/µL for women)

**Fig. 2:**
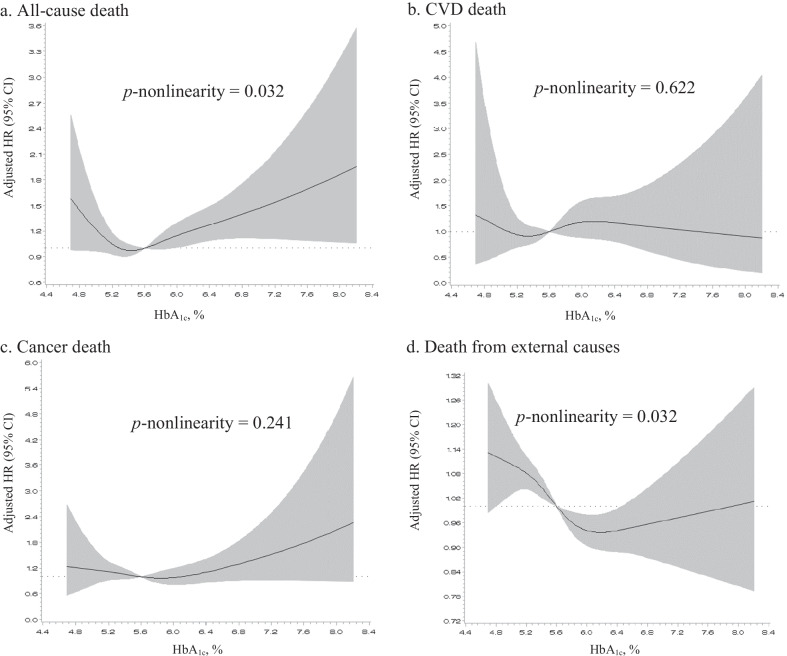
Risk of death according to restricted cubic spline regression among participants without known diabetes. The data were adjusted for age, sex, residential area, body mass index, smoking, alcohol use, regular exercise, education, hypertension, and dyslipidaemia

Restricted cubic spline regression models for individuals without known diabetes revealed a J-shaped association between continuous baseline HbA1c levels and all-cause mortality (*p*-nonlinearity = 0.032) (Fig. 2a). However, no pattern was observed in the associations between HbA1c and mortality from CVD and external causes (Fig. 2b, d). A slight J-shaped association was observed in cancer mortality, but it was not significant (Fig. 2c). When we analyzed the predictive value of HbA1c for mortality using ROC, the performance was slightly increased for cancer mortality compared with the conventional model (Additional file 3).

## Discussion

In the present study of the general Korean population with neither a history of cancer nor CVD, non-diabetic adults with low HbA1c were at significantly higher risk of all-cause and cancer mortality compared with individuals with HbA1c of 5.5–5.9%. Higher HbA1c levels in the prediabetic and diabetic ranges were associated with an increased risk of all-cause mortality in a dose–response manner. HbA1c levels lower than diabetic range was not associated with increased risk of CVD mortality. Adults with known diabetes had higher risk of all-cause, CVD, and cancer mortality. Death from external causes, a negative control, was not associated with either low or high HbA1c. Furthermore, when stratified by the median RBC count or by having liver diseases, there was a strong association between low HbA1c levels and all-cause mortality among individuals with low RBC counts or liver diseases.

Our results are consistent with previous findings of U-shaped or J-shaped associations between HbA1c levels and mortality [12–16]. In the Atherosclerosis Risk in Communities (ARIC) study, non-diabetic participants with HbA1c levels < 5.0% (HR, 1.48; 95% CI 1.21–1.81), as well as those with HbA1c levels ≥ 5.5%, were at a significantly increased risk of death from any cause compared with those with HbA1c 5.0 to < 5.5% [15]. In the general German population without known diabetes, restricted cubic spline models showed a U-shaped association between HbA1c levels and all-cause mortality: the lowest risk was at HbA1c levels of 5.4–5.6% and a significantly increased risk were observed both at ≤ 5.0% and ≥ 6.4% [16]. The association between extremely low HbA1c level and mortality was assessed among US National Health and Nutrition Study III participants without diabetes. Compared with HbA1c levels of 5.0–5.4%, HbA1c levels < 4.0% were associated with significantly increased risk of all-cause mortality in a fully adjusted model (HR, 2.90; 95% CI 1.25–6.76) [12]. In the general New Zealand population, the HR for all-cause mortality among individuals with HbA1c levels < 4.0% versus 4.0–4.9% was higher with marginal statistical significance (HR, 2.90; 95% CI 0.91–9.19) [13]. In the current study, we were unable to examine extremely low HbA1c levels < 4.0% because the number of participants in that group was very small (*n* = 2).

On the other hand, several prospective cohort studies have reported that only high HbA1c levels increase mortality [9–11, 17]. In the general Japanese population, compared with HbA1c levels < 5.0%, high HbA1c levels (> 6.0%) in individuals without treatment for diabetes were significantly associated with an increased risk of all-cause and CVD mortality [9]. In Singaporean Chinese adults without diagnosed diabetes, compared with HbA1c levels of 5.4–5.6%, only HbA1c levels ≥ 6.5% were significantly associated with all-cause (HR, 1.96; 95% CI 1.56–2.46), CVD (HR, 2.63; 95% CI 1.77–3.90), and cancer (HR, 1.51; 95% CI 1.04–2.18) mortality [17]. In Australian adults (aged ≥ 25 years) without diagnosed diabetes, HbA1c levels exhibited a linear relationship with all-cause and CVD mortality [11].

Biological mechanisms between hyperglycaemia and an increased risk of mortality may be due to the vascular damage caused by increased oxidative stress and endothelial dysfunction in individuals with impaired fasting glucose or impaired glucose tolerance [23, 24]. Moreover, elevated HbA1c levels influences cancer progression through an increase in the levels of insulin, insulin-like growth factor-1 (IGF-1), and inflammatory cytokines in circulation [25, 26]. However, the potential mechanism underlying the association between low HbA1c levels and increased mortality remains unclear. Low HbA1c levels have been correlated with impaired RBC related indices and increased liver function indices [12, 22]. These factors, in turn, were shown to correlate with inflammatory processes and increased morbidity and mortality [27, 28]. In other words, low HbA1c levels are considered a marker of deteriorated health condition. Thus, the association between low HbA1c levels and higher mortality may be explained by a result of reverse causation due to comorbid conditions [12, 22]. In our study sample, participants with HbA1c levels < 5% had lower RBC, haemoglobin, and haematocrit levels and higher total bilirubin levels and prevalence of liver diseases than did those with HbA1c levels of 5.5–5.9%, the reference group. Moreover, HbA1c levels < 5% were associated with an increased risk of all-cause mortality only among people with pre-existing liver disease or less than median RBC counts. These results suggest potential roles of RBC and liver function in the association between low HbA1c levels and increased mortality.

The strength of this study lies in the long-term population-based cohort design (16-year follow-up) and the consideration of changes of HbA1c levels and other clinical and lifestyle variables during the follow-up period. Most of the previous studies used the time-fixed method with the baseline HbA1c levels and did not reflect the change of HbA1c during the follow-up period [9–17]. However, we used the time-dependent method considering the change of HbA1c over time which is more reflective of clinical practice. To the best of our knowledge, this is the first study to evaluate the association between HbA1c levels and mortality in the general Korean population and to find an increased risk of mortality at low HbA1c levels in the Asian population.

Despite its strengths, the current study had some limitations. First, the participants were aged 40–70 years at the baseline examination and were enrolled from two communities. Therefore, the findings of this study may not be directly generalizable to younger adults or the entire Korean population. However, general characteristics and HbA1c level distribution were similar when we compared our study population with the Korea National Health and Nutrition Examination Survey (KNHANES) participants, who are a representative sample. Specifically, among the KNHANES participants aged 40–70 in 2019, 44% were men, the mean age was 55.0 years, and the mean HbA1c level was 5.9% [29]. Second, among the 9294 participants who met the inclusion criteria at baseline, 14.5% did not attend any follow-up examination until 2016. However, the major variables, such as HbA1c levels, age, sex, and comorbidities, were similar between participants and nonparticipants in the follow-up examinations (data not shown). Therefore, it is unlikely that the association between HbA1c levels and mortality found in the time-dependent analysis using follow-up data is severely underestimated or overestimated. Third, a small number of participants with extremely low HbA1c levels in our study sample hindered more detailed analysis. Fourth, information on the diagnosis and treatment of diabetes, hypertension, dyslipidaemia, and liver diseases was obtained from questionnaires. Participants omitting to report existing diseases or having undiagnosed health conditions could have limited our ability to classify people with diabetes or other existing diseases. However, we collected data on the disease history using a structured questionnaire with questions about each disease asked separately by trained interviewers. Moreover, missing rates of related variables were very low (0.04%). Finally, although we adjusted for several potential confounders in our analysis, residual confounding may remain due to unmeasured or uncontrolled confounders such as statins use that might affect blood glucose control [30].

## Conclusions

In summary, there was a U-shaped association between HbA1c levels at baseline and over time and all-cause mortality in middle-aged and older Koreans. Additionally, we found increase risk of cancer mortality both in low and high HbA1c groups, but the risk of CVD mortality was increased only in the high HbA1c group over time. These findings suggest that people with low HbA1c levels, as well as those with high HbA1c levels, have an increased risk of mortality. HbA1c testing may improve the identification of individuals with a high risk of mortality. In particular, for people with liver diseases and low HbA1c levels, more careful management is suggested to identify any deteriorating health conditions. Causal mechanisms underlying the increased cause-specific mortality risk in the lower HbA1c range warrant investigation. Thus, further studies with a large number of individuals with very low HbA1c levels and a detailed assessment of morbid conditions are needed. In addition, further studies to identify the usefulness and appropriate cut-off HbA1c level for predicting mortality are needed.

## Supplementary Information

**Additional file 1.** Risk of death according to HbA_1c_ levels at baseline or over time when further adjusted for covariates. Model 1: Adjusted for age, sex, residential area, body mass index, smoking, alcohol use, regular exercise, education, hypertension, dyslipidaemia, and RBC count. Model 2: Adjusted for age, sex, residential area, body mass index, smoking, alcohol use, regular exercise, education, hypertension, dyslipidaemia, and haemoglobin. Model 3: Adjusted for age, sex, residential area, body mass index, smoking, alcohol use, regular exercise, education, hypertension, dyslipidaemia, and anaemia. Model 4: Adjusted for age, sex, residential area, body mass index, smoking, alcohol use, regular exercise, education, hypertension, dyslipidaemia, and liver diseases. *RBC* red blood cell.

**Additional file 2.** Mortality rate and risk of death according to HbA1c levels at baseline or over time after excluding those who died within the first 2 years of follow-up (*N* = 9217). *Adjusted for age, sex, residential area, body mass index, smoking, alcohol use, regular exercise, education, hypertension, and dyslipidaemia.

**Additional file 3.** Comparison of ROC curves and AUC areas of the conventional model* and the model plus HbA1c. *Model with age, sex, residential area, body mass index, smoking, alcohol use, regular exercise, education, hypertension, and dyslipidemia.

## Data Availability

The KoGES data and biospecimens are available for research purposes from https://www.nih.go.kr/ on reasonable request.

## Abbreviations

ALT: Alanine aminotransferase
AST: Aspartate aminotransferase
BMI: Body mass index
CI: Confidence interval
CVD: Cardiovascular disease
DBP: Diastolic blood pressure
HbA_1c_: Haemoglobin A1C
HDL: High-density lipoprotein
HR: Hazard ratio
KoGES: Korean Genome and Epidemiology Study
RBC: Red blood cell
SBP: Systolic blood pressure
