# Supplementary material for: Association between haemoglobin A1c and all-cause and cause-specific mortality in middle-aged and older Koreans: a prospective cohort study
Source: Nutr Metab (Lond). 2022 Jul 14;19:46. doi: 10.1186/s12986-022-00682-4 (PMC9284843; doi:10.1186/s12986-022-00682-4)
Supplement: Supplementary file 1 — Additional file 1. Risk of death according to HbA1c levels at baseline or over time when further adjusted for covariates. Model 1: Adjusted for age, sex, residential area, body mass index, smoking, alcohol use, regular exercise, education, hypertension, dyslipidaemia, and RBC count. Model 2: Adjusted for age, sex, residential area, body mass index, smoking, alcohol use, regular exercise, education, hypertension, dyslipidaemia, and haemoglobin. Model 3: Adjusted for age, sex, residential area, body mass index, smoking, alcohol use, regular exercise, education, hypertension, dyslipidaemia, and anaemia. Model 4: Adjusted for age, sex, residential area, body mass index, smoking, alcohol use, regular exercise, education, hypertension, dyslipidaemia, and liver diseases. RBC red blood cell. [file 12986_2022_682_MOESM1_ESM.docx]

**Additional File 1. Risk of death according to HbA_1c_ levels at baseline or over time when further adjusted for covariates**

|  | HbA_1c_ in participants without known diabetes | | | | | Known diabetes |
| --- | --- | --- | --- | --- | --- | --- |
|  | <5.0% | 5.0–5.4% | 5.5–5.9% | 6.0–6.4% | ≥6.5% |  |
| **HbA_1c_ at baseline*, n*** | 303 | 2962 | 3930 | 1038 | 457 | 604 |
| All-cause death*, n* | 28 | 228 | 337 | 139 | 69 | 143 |
| Model 1 (Adjusted HR (95% CI)) | 1.59 (1.08–2.35) | 1.16 (0.98–1.37) | ref. | 1.51 (1.23–1.84) | 1.65 (1.27–2.15) | 2.30 (1.88–2.81) |
| Model 2 (Adjusted HR (95% CI)) | 1.72 (1.17–2.54) | 1.20 (1.01–1.43) | ref. | 1.48 (1.22–1.81) | 1.62 (1.24–2.11) | 2.31 (1.89–2.82) |
| Model 3 (Adjusted HR (95% CI)) | 1.71 (1.16–2.53) | 1.20 (1.02–1.43) | ref. | 1.51 (1.23–1.84) | 1.63 (1.25–2.12) | 2.29 (1.87–2.80) |
| Model 4 (Adjusted HR (95% CI)) | 1.71 (1.16–2.53) | 1.20 (1.01–1.42) | ref. | 1.50 (1.23–1.83) | 1.63 (1.25–2.12) | 2.21 (1.81–2.71) |
| CVD death*, n* | 3 | 33 | 68 | 30 | 15 | 36 |
| Model 1 (Adjusted HR (95% CI)) | 1.06 (0.32–3.46) | 0.99 (0.65–1.51) | ref. | 1.38 (0.89–2.15) | 1.41 (0.80–2.49) | 2.31 (1.51–3.55) |
| Model 2 (Adjusted HR (95% CI)) | 1.07 (0.33–3.47) | 0.99 (0.65–1.51) | ref. | 1.38 (0.89–2.14) | 1.41 (0.80–2.48) | 2.32 (1.51–3.55) |
| Model 3 (Adjusted HR (95% CI)) | 1.07 (0.33–3.46) | 0.99 (0.65–1.51) | ref. | 1.38 (0.89–2.14) | 1.41 (0.80–2.48) | 2.31 (1.51–3.54) |
| Model 4 (Adjusted HR (95% CI)) | 1.08 (0.33–3.48) | 0.99 (0.65–1.51) | ref. | 1.38 (0.89–2.14) | 1.41 (0.80–2.48) | 2.33 (1.52–3.57) |
| Cancer death*, n* | 12 | 107 | 126 | 52 | 27 | 35 |
| Model 1 (Adjusted HR (95% CI)) | 1.57 (0.85–2.90) | 1.39 (1.07–1.80) | ref. | 1.44 (1.04–2.00) | 1.68 (1.10–2.57) | 1.40 (0.96–2.05) |
| Model 2 (Adjusted HR (95% CI)) | 1.75 (0.96–3.21) | 1.44 (1.11–1.87) | ref. | 1.42 (1.03–1.97) | 1.64 (1.07–2.52) | 1.41 (0.96–2.06) |
| Model 3 (Adjusted HR (95% CI)) | 1.78 (0.97–3.25) | 1.44 (1.11–1.87) | ref. | 1.44 (1.04–2.00) | 1.65 (1.08–2.53) | 1.40 (0.96–2.05) |
| Model 4 (Adjusted HR (95% CI)) | 1.70 (0.94–3.07) | 1.43 (1.10–1.85) | ref. | 1.44 (1.04–2.00) | 1.67 (1.09–2.56) | 1.31 (0.89–1.92) |
| Death from external causes, *n* | 3 | 38 | 50 | 19 | 6 | 9 |
| Model 1 (Adjusted HR (95% CI)) | 0.76 (0.22–2.68) | 1.10 (0.72–1.69) | ref. | 1.38 (0.80–2.37) | 1.03 (0.43–2.48) | 0.97 (0.47–1.99) |
| Model 2 (Adjusted HR (95% CI)) | 0.90 (0.27–3.00) | 1.14 (0.75–1.75) | ref. | 1.35 (0.78–2.31) | 1.01 (0.42–2.45) | 1.00 (0.49–2.04) |
| Model 3 (Adjusted HR (95% CI)) | 0.91 (0.28–2.99) | 1.14 (0.74–1.75) | ref. | 1.37 (0.79–2.35) | 1.02 (0.42–2.46) | 0.99 (0.48–2.03) |
| Model 4 (Adjusted HR (95% CI)) | 0.94 (0.29–3.09) | 1.13 (0.74–1.74) | ref. | 1.36 (0.79–2.34) | 1.03 (0.43–2.48) | 1.01 (0.49–2.07) |
| **HbA_1c_ over time** |  |  | 0 |  |  |  |
| All-cause death |  |  |  |  |  |  |
| Model 1 (Adjusted HR (95% CI)) | 1.59 (1.16–2.17) | 1.09 (0.92–1.30) | ref. | 1.32 (1.05–1.64) | 1.42 (1.01–2.01) | 1.84 (1.53–2.21) |
| Model 2 (Adjusted HR (95% CI)) | 1.79 (1.32–2.44) | 1.14 (0.96–1.36) | ref. | 1.27 (1.02–1.59) | 1.36 (0.96–1.92) | 1.88 (1.57–2.26) |
| Model 3 (Adjusted HR (95% CI)) | 1.79 (1.31–2.44) | 1.14 (0.96–1.36) | ref. | 1.29 (1.03–1.61) | 1.37 (0.97–1.94) | 1.90 (1.59–2.28) |
| Model 4 (Adjusted HR (95% CI)) | 1.80 (1.32–2.46) | 1.13 (0.95–1.35) | ref. | 1.31 (1.04–1.63) | 1.39 (0.98–1.97) | 2.01 (1.67–2.40) |
| CVD death |  |  |  |  |  |  |
| Model 1 (Adjusted HR (95% CI)) | 1.49 (0.63–3.52) | 1.16 (0.76–1.79) | ref. | 1.44 (0.87–2.37) | 2.40 (1.28–4.52) | 2.24 (1.49–3.38) |
| Model 2 (Adjusted HR (95% CI)) | 1.52 (0.64–3.59) | 1.17 (0.76–1.81) | ref. | 1.43 (0.87–2.35) | 2.39 (1.27–4.48) | 2.25 (1.49–3.38) |
| Model 3 (Adjusted HR (95% CI)) | 1.52 (0.64–3.61) | 1.17 (0.76–1.80) | ref. | 1.43 (0.87–2.36) | 2.39 (1.27–4.48) | 2.27 (1.51–3.42) |
| Model 4 (Adjusted HR (95% CI)) | 1.52 (0.64–3.61) | 1.17 (0.76–1.80) | ref. | 1.43 (0.87–2.36) | 2.39 (1.28–4.49) | 2.28 (1.51–3.44) |
| Cancer death |  |  |  |  |  |  |
| Model 1 (Adjusted HR (95% CI)) | 1.79 (1.15–2.80) | 1.18 (0.91–1.54) | ref. | 1.09 (0.75–1.59) | 0.74 (0.36–1.53) | 1.41 (1.04–1.91) |
| Model 2 (Adjusted HR (95% CI)) | 2.10 (1.34–3.29) | 1.25 (0.95–1.63) | ref. | 1.05 (0.72–1.52) | 0.71 (0.34–1.46) | 1.46 (1.08–1.97) |
| Model 3 (Adjusted HR (95% CI)) | 2.14 (1.37–3.32) | 1.24 (0.95–1.62) | ref. | 1.07 (0.73–1.55) | 0.71 (0.34–1.47) | 1.48 (1.10–2.00) |
| Model 4 (Adjusted HR (95% CI)) | 2.09 (1.35–3.24) | 1.23 (0.94–1.60) | ref. | 1.09 (0.75–1.58) | 0.73 (0.36–1.52) | 1.55 (1.15–2.09) |
| Death from external causes |  |  |  |  |  |  |
| Model 1 (Adjusted HR (95% CI)) | 0.64 (0.20–2.12) | 1.21 (0.78–1.87) | ref. | 1.58 (0.90–2.77) | 0.78 (0.24–2.55) | 1.26 (0.71–2.24) |
| Model 2 (Adjusted HR (95% CI)) | 0.68 (0.21–2.23) | 1.23 (0.79–1.90) | ref. | 1.56 (0.88–2.74) | 0.77 (0.24–2.52) | 1.28 (0.72–2.28) |
| Model 3 (Adjusted HR (95% CI)) | 0.68 (0.21–2.20) | 1.24 (0.80–1.91) | ref. | 1.56 (0.89–2.75) | 0.77 (0.24–2.52) | 1.24 (0.70–2.22) |
| Model 4 (Adjusted HR (95% CI)) | 0.68 (0.21–2.22) | 1.22 (0.79–1.90) | ref. | 1.57 (0.89–2.77) | 0.78 (0.24–2.54) | 1.31 (0.73–2.32) |

Model 1: Adjusted for age, sex, residential area, body mass index, smoking, alcohol use, regular exercise, education, hypertension, dyslipidemia, and RBC count.

Model 2: Adjusted for age, sex, residential area, body mass index, smoking, alcohol use, regular exercise, education, hypertension, dyslipidemia, and haemoglobin.

Model 3: Adjusted for age, sex, residential area, body mass index, smoking, alcohol use, regular exercise, education, hypertension, dyslipidemia, and anaemia.

Model 4: Adjusted for age, sex, residential area, body mass index, smoking, alcohol use, regular exercise, education, hypertension, dyslipidemia, and liver diseases.

RBC, red blood cell
