# Supplementary material for: Association between haemoglobin A1c and all-cause and cause-specific mortality in middle-aged and older Koreans: a prospective cohort study
Source: Nutr Metab (Lond). 2022 Jul 14;19:46. doi: 10.1186/s12986-022-00682-4 (PMC9284843; doi:10.1186/s12986-022-00682-4)
Supplement: Supplementary file 2 — Additional file 2. Mortality rate and risk of death according to HbA1c levels at baseline or over time after excluding those who died within the first 2 years of follow-up (N = 9217). *Adjusted for age, sex, residential area, body mass index, smoking, alcohol use, regular exercise, education, hypertension, and dyslipidaemia. [file 12986_2022_682_MOESM2_ESM.docx]

| **Additional File 2. Mortality rate and risk of death according to HbA1c levels at baseline or over time after excluding those who died within the first two years of follow-up (N=9,217)** | | | | | | | | | | | | | | | | | | | | | |
| --- | --- | --- | --- | --- | --- | --- | --- | --- | --- | --- | --- | --- | --- | --- | --- | --- | --- | --- | --- | --- | --- |
|  | HbA1c in participants without known diabetes | | | | | | | | | | | | | | | | | Known diabetes | | | |
|  | <5.0% | | | | 5.0-5.4% | | | | 5.5-5.9% | 6.0-6.4% | | | | ≥6.5% | | | |  |  |  |  |
| **HbA1c at baseline, n** | 298 | | | | 2945 | | | | 3900 | 1029 | | | | 451 | | | | 594 | | | |
| Person-years of follow-up | 4539 | | | | 44948 | | | | 59595 | 15449 | | | | 6764 | | | | 8589 | | | |
| All-cause death, n | 23 | | | | 211 | | | | 307 | 130 | | | | 63 | | | | 133 | | | |
| Mortality (per 1,000 person-years) | 5.1 | | | | 4.7 | | | | 5.2 | 8.4 | | | | 9.3 | | | | 15.5 | | | |
| Adjusted HR (95% CI)* | 1.60 | (1.05 | - | 2.46) | 1.23 | (1.03 | - | 1.47) | ref. | 1.54 | (1.25 | - | 1.89) | 1.61 | (1.22 | - | 2.12) | 2.35 | (1.91 | - | 2.90) |
| CVD death, n | 2 | | | | 30 | | | | 62 | 28 | | | | 12 | | | | 33 | | | |
| Mortality (per 1,000 person-years) | 0.4 | | | | 0.7 | | | | 1.0 | 1.8 | | | | 1.8 | | | | 3.8 | | | |
| Adjusted HR (95% CI)* | 0.80 | (0.19 | - | 3.36) | 1.00 | (0.65 | - | 1.55) | ref. | 1.41 | (0.89 | - | 2.23) | 1.22 | (0.66 | - | 2.28) | 2.38 | (1.52 | - | 3.75) |
| Cancer death, n | 10 | | | | 102 | | | | 114 | 51 | | | | 26 | | | | 31 | | | |
| Mortality (per 1,000 person-years) | 2.2 | | | | 2.3 | | | | 1.9 | 3.3 | | | | 3.8 | | | | 3.6 | | | |
| Adjusted HR (95% CI)* | 1.68 | (0.87 | - | 3.22) | 1.52 | (1.16 | - | 1.99) | ref. | 1.54 | (1.10 | - | 2.15) | 1.74 | (1.13 | - | 2.69) | 1.37 | (0.92 | - | 2.04) |
| Death from external causes, n | 3 | | | | 33 | | | | 43 | 14 | | | | 4 | | | | 8 | | | |
| Mortality (per 1,000 person-years) | 0.7 | | | | 0.7 | | | | 0.7 | 0.9 | | | | 0.6 | | | | 0.9 | | | |
| Adjusted HR (95% CI)* | 1.11 | (0.34 | - | 3.69) | 1.16 | (0.73 | - | 1.83) | ref. | 1.15 | (0.62 | - | 2.11) | 0.79 | (0.27 | - | 2.26) | 1.08 | (0.50 | - | 2.31) |
| **HbA1c over time,** adjusted HR (95% CI)* | |  |  |  |  |  |  |  |  |  |  |  |  |  |  |  |  |  |  |  |  |
| All-cause death | 1.78 | (1.28 | - | 2.46) | 1.15 | (0.96 | - | 1.39) | ref. | 1.32 | (1.05 | - | 1.67) | 1.31 | (0.90 | - | 1.91) | 2.06 | (1.71 | - | 2.49) |
| CVD death | 1.37 | (0.53 | - | 3.51) | 1.20 | (0.76 | - | 1.89) | ref. | 1.47 | (0.87 | - | 2.50) | 2.18 | (1.08 | - | 4.41) | 2.31 | (1.50 | - | 3.56) |
| Cancer death | 2.20 | (1.39 | - | 3.50) | 1.29 | (0.98 | - | 1.70) | ref. | 1.16 | (0.79 | - | 1.70) | 0.70 | (0.32 | - | 1.51) | 1.60 | (1.17 | - | 2.18) |
| Death from external causes | 0.76 | (0.23 | - | 2.52) | 1.25 | (0.78 | - | 2.01) | ref. | 1.35 | (0.71 | - | 2.58) | 0.32 | (0.04 | - | 2.33) | 1.37 | (0.75 | - | 2.50) |
| * Adjusted for age, sex, residential area, body mass index, smoking, alcohol use, regular exercise, education, hypertension, and dyslipidemia. | | | | | | | | | | | | | | | | | | | | | |
