# Supplementary material for: Association between haemoglobin A1c and all-cause and cause-specific mortality in middle-aged and older Koreans: a prospective cohort study
Source: Nutr Metab (Lond). 2022 Jul 14;19:46. doi: 10.1186/s12986-022-00682-4 (PMC9284843; doi:10.1186/s12986-022-00682-4)
Supplement: Supplementary file 3 — Additional file 3. Comparison of ROC curves and AUC areas of the conventional model* and the model plus HbA1c. *Model with age, sex, residential area, body mass index, smoking, alcohol use, regular exercise, education, hypertension, and dyslipidemia. [file 12986_2022_682_MOESM3_ESM.docx]

**Additional File 3. Comparison of ROC curves and AUC areas of the conventional model* and the model plus HbA1c**

a. All-cause death b. CVD death


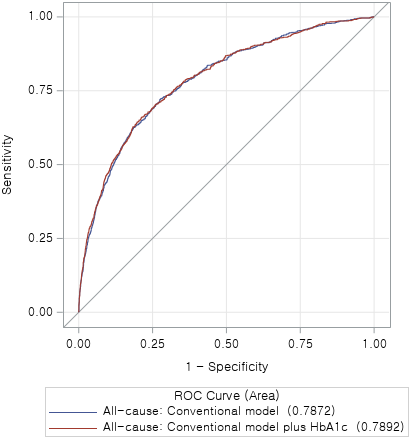

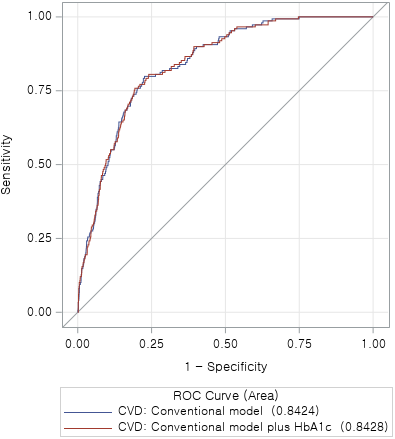


Difference = 0.0004

*p*-value = 0.778

Difference = 0.002

*p*-value = 0.150

c. Cancer death d. Death from external causes


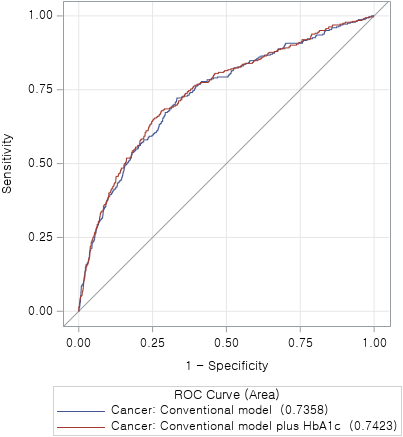

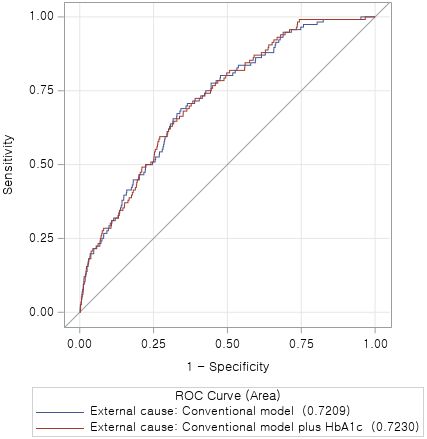


Difference = 0.002

*p*-value = 0.584

Difference = 0.006

*p*-value = 0.043

^*^ The conventional model included age, sex, residential area, body mass index, smoking, alcohol use, regular exercise, education, hypertension, and dyslipidemia.
